# Supplementary material for: BRAF/EZH2 Signaling Represses miR-129-5p Inhibition of SOX4 Thereby Modulating BRAFi Resistance in Melanoma
Source: Cancers (Basel). 2021 May 15;13(10):2393. doi: 10.3390/cancers13102393 (PMC8155874; doi:10.3390/cancers13102393)
Supplement: Supplementary file 1 [file cancers-13-02393-s001.zip › cancers-1197471supplementary.pdf]

Supplemental Figures

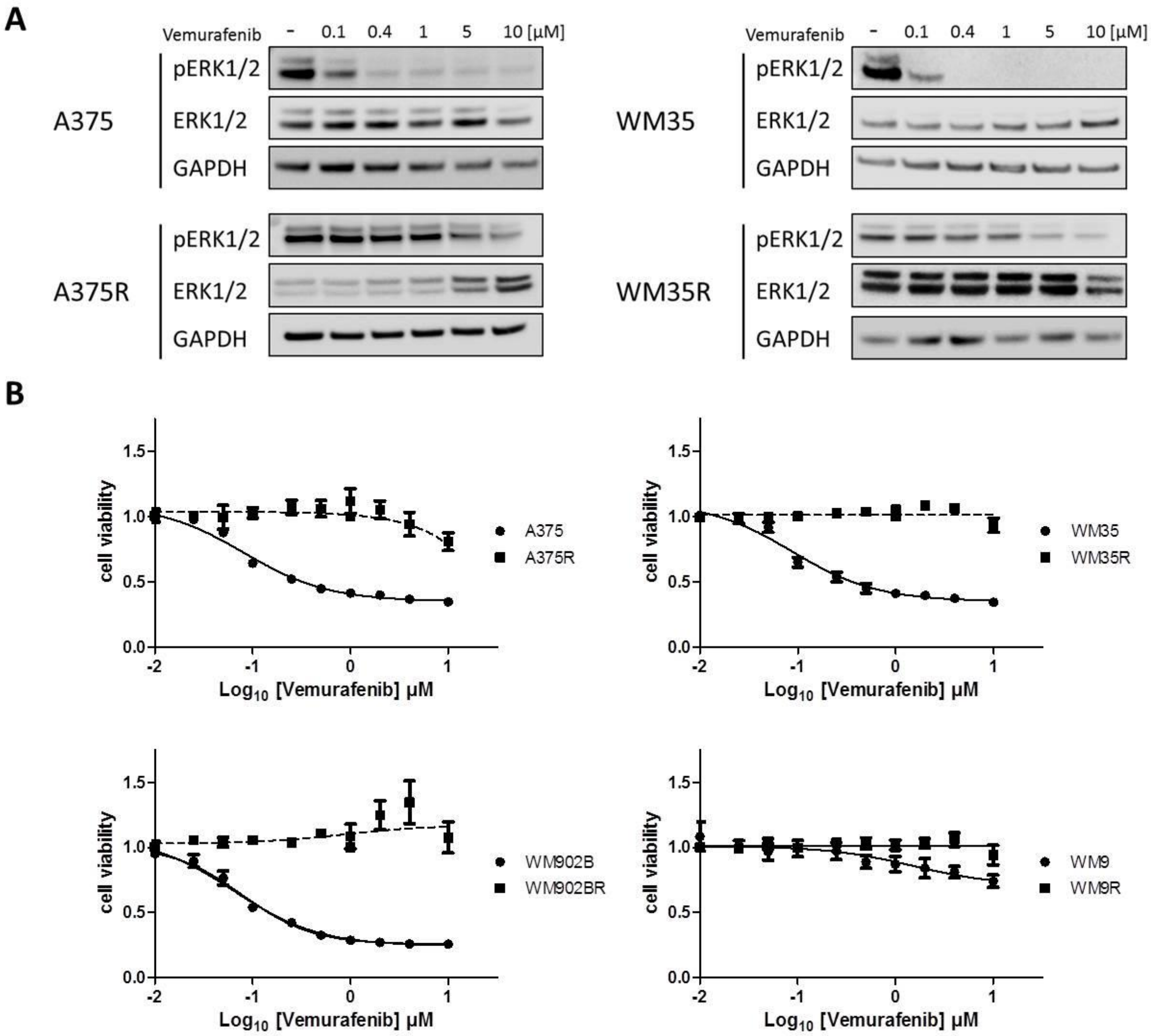

Figure S1. Establishment of Vemurafenib resistant cell lines. (A) Western blot analyses of phospho ERK 1/2 and total ERK 1/2 after Vemurafenib treatment in parental and resistant BRAF mutated melanoma cell lines (A375, WM35). GAPDH was used as loading control. (B) Cell Viability Assay of parental melanoma cell lines and their corresponding resistant clone after 72 h treatment with increasing Vemurafenib concentrations.

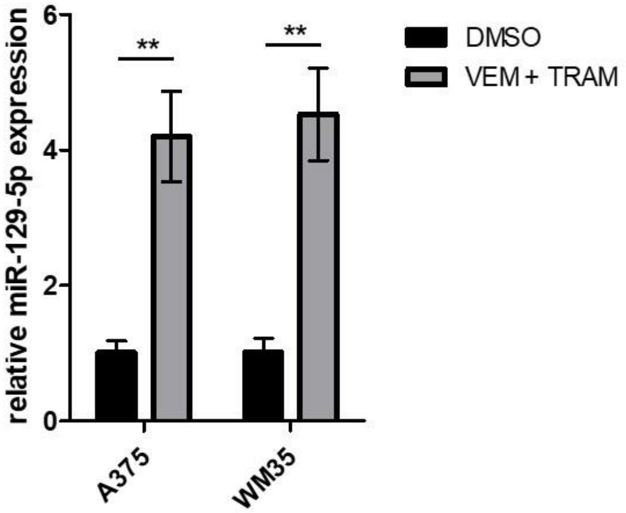

Figure S2. miR-129-5p expression is increased after BRAFi/MEKi combinatory treatment. qRT-PCR analyses of miR-129-5p expression in A375 and WM35 cells after combinatory treatment with Vemurafenib (0.4 μM) and Trametinib (10 nM) or DMSO (control) for 24 h. **.**

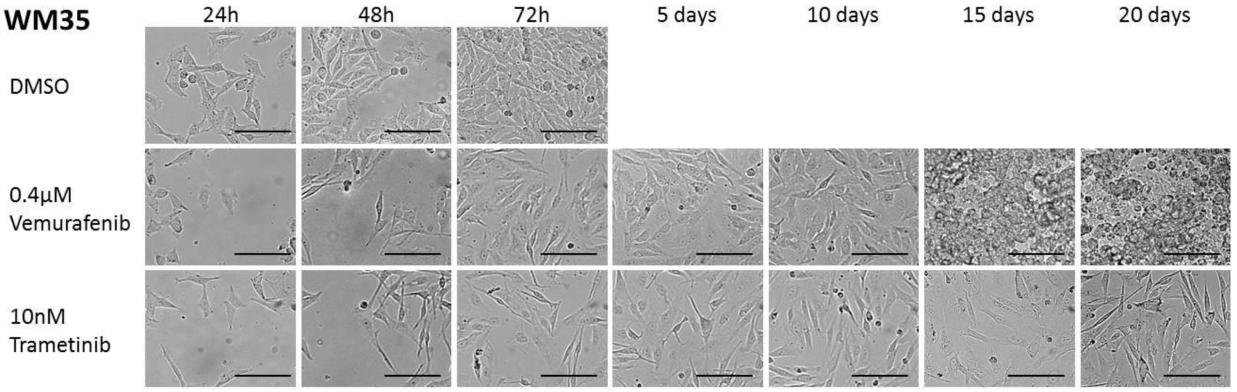

Figure S3. Cell morphology of WM35 during long term BRAFi and MEKi treatment. (A) WM35 cells were treated with Vemurafenib or Trametinib every second day for a period of 20 days. Cells were imaged at the mentioned time points. Scale bar represents 200 μm.

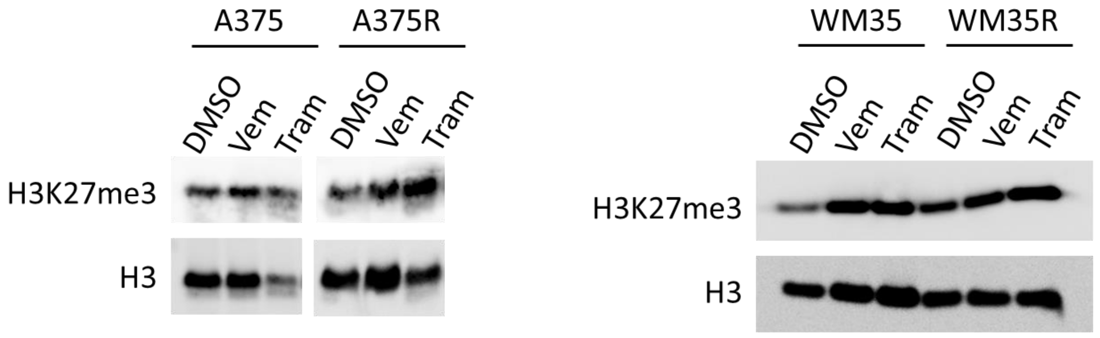

Figure S4. H3K27me3 after BRAFi and MEKi. Western blot analyses of H3K27me3 and H3 in A375, A375R, WM35 and WM35R cell lines. Cells were treated with DMSO, 0.4 μM Vemurafenib (Vem) or 10 nM Trametinib (Tram) for 48 h.

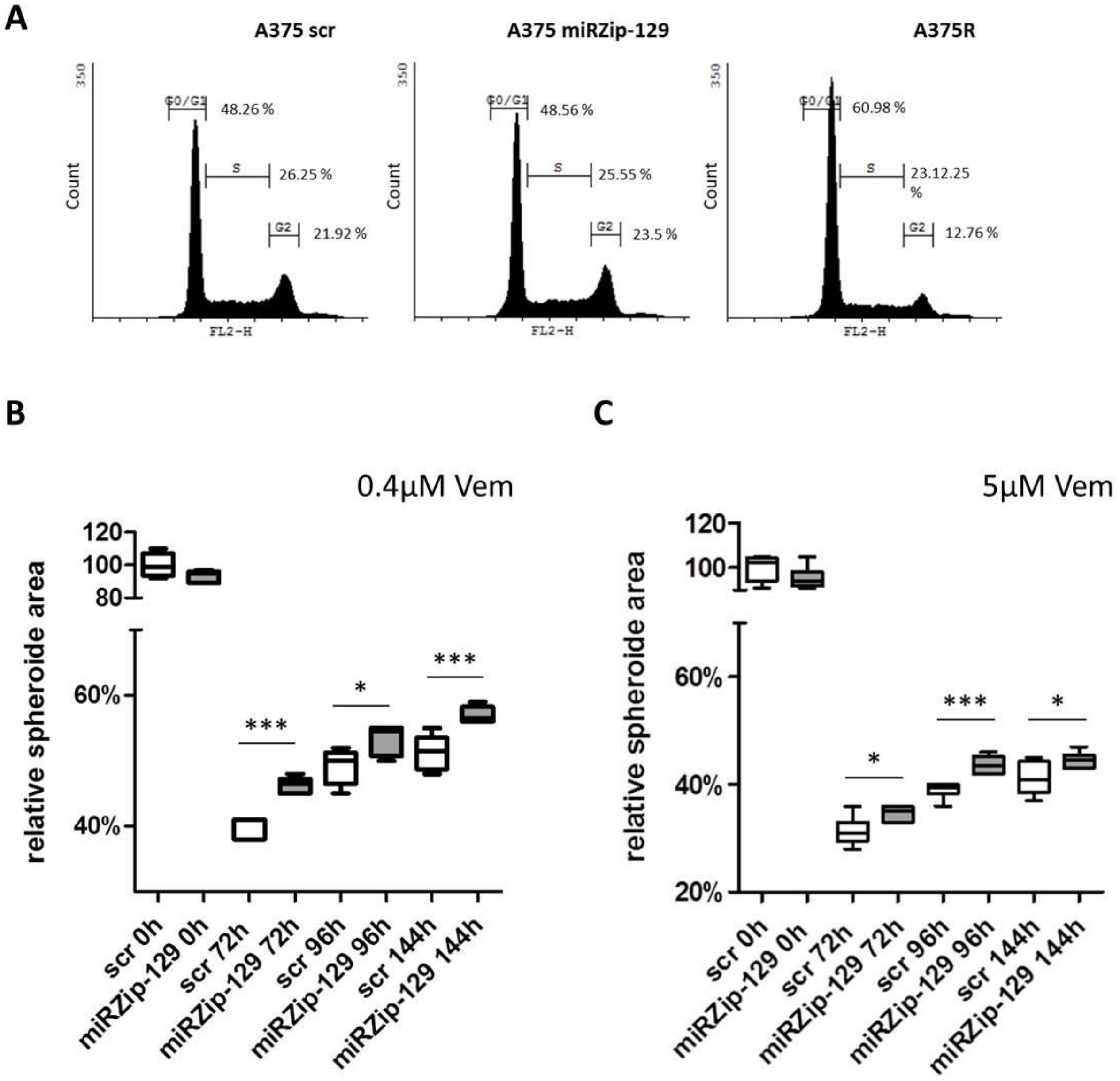

Figure S5. miR-129-5p acts as tumor suppressor. (A) Cell cycle analysis of DMSO treated A375 cells transfected with miRZip-scr. or miRZIP-129 construct in comparison to BRAFi resistant A375R cells. Growth analyses of 3D spheroid cultures were assessed 96 h after seeding and a following Vemurafenib treatment, at 0.4 µM (B) or 5 µM (C), for 72 h, 96 h and 144 h. Spheroid area of at least 5 spheroids was measured using ImageJ software.
