# Supplementary material for: BRAF/EZH2 Signaling Represses miR-129-5p Inhibition of SOX4 Thereby Modulating BRAFi Resistance in Melanoma
Source: Cancers (Basel). 2021 May 15;13(10):2393. doi: 10.3390/cancers13102393 (PMC8155874; doi:10.3390/cancers13102393)

Figure 3A

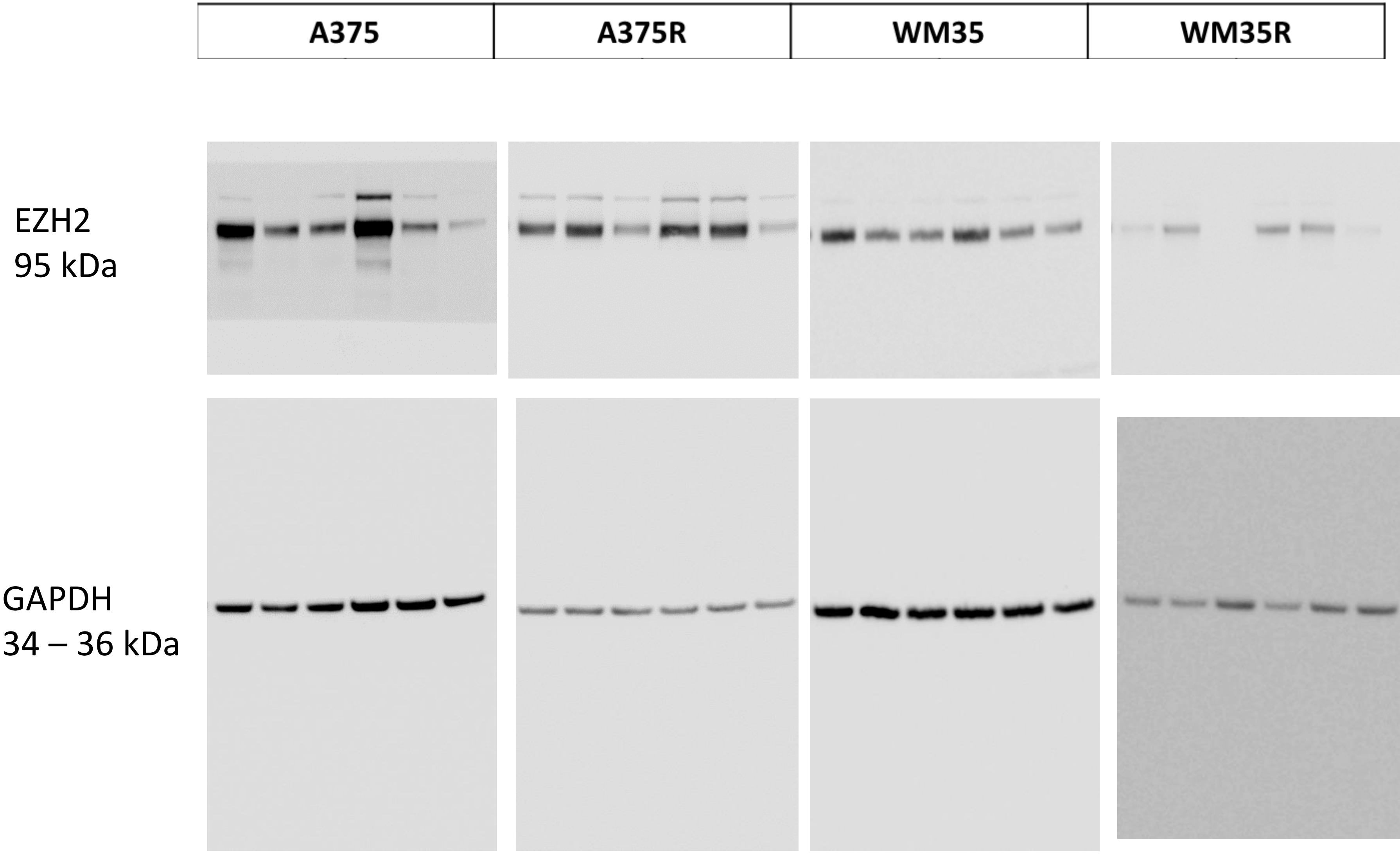

Figure 5C

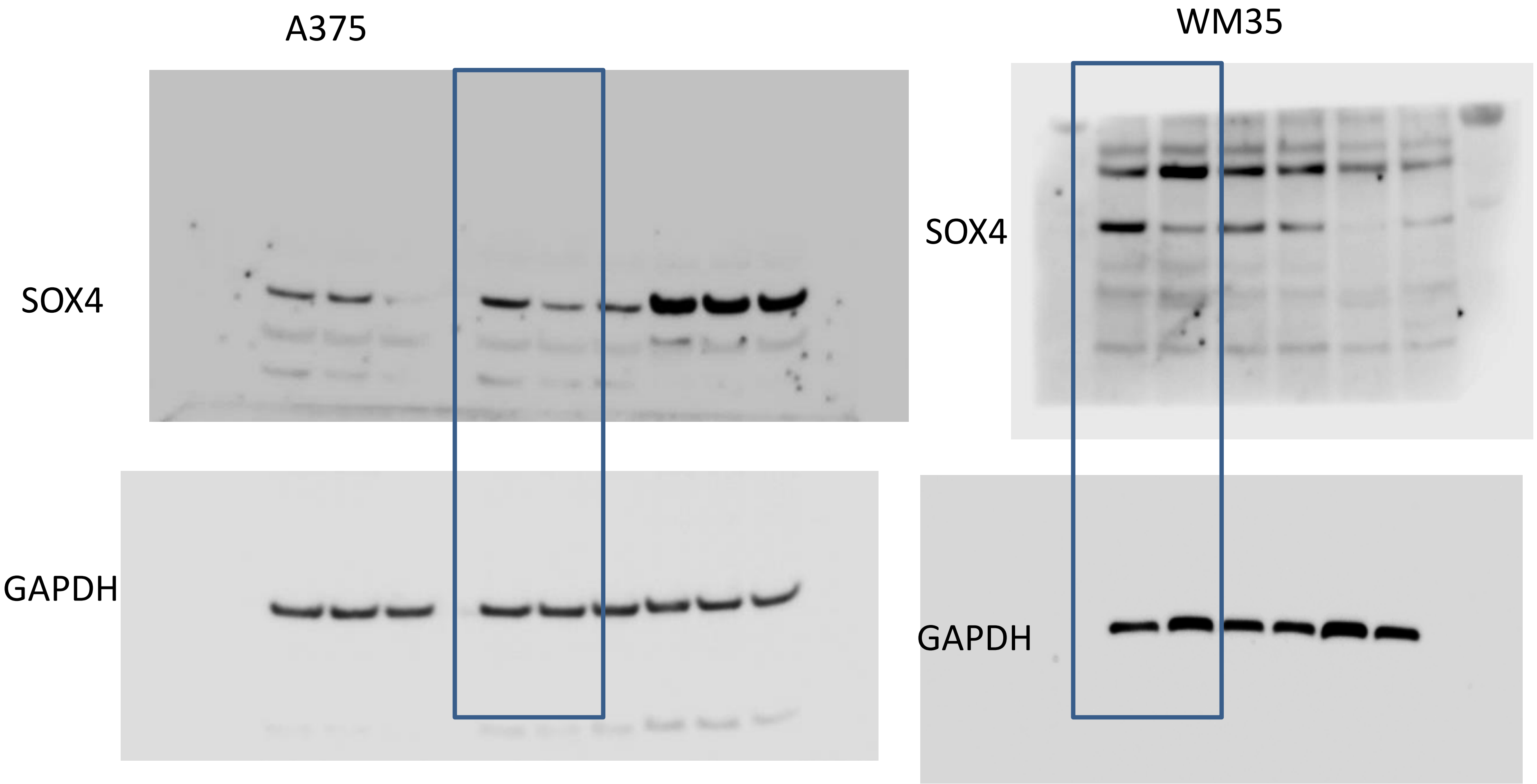

Figure 5 D

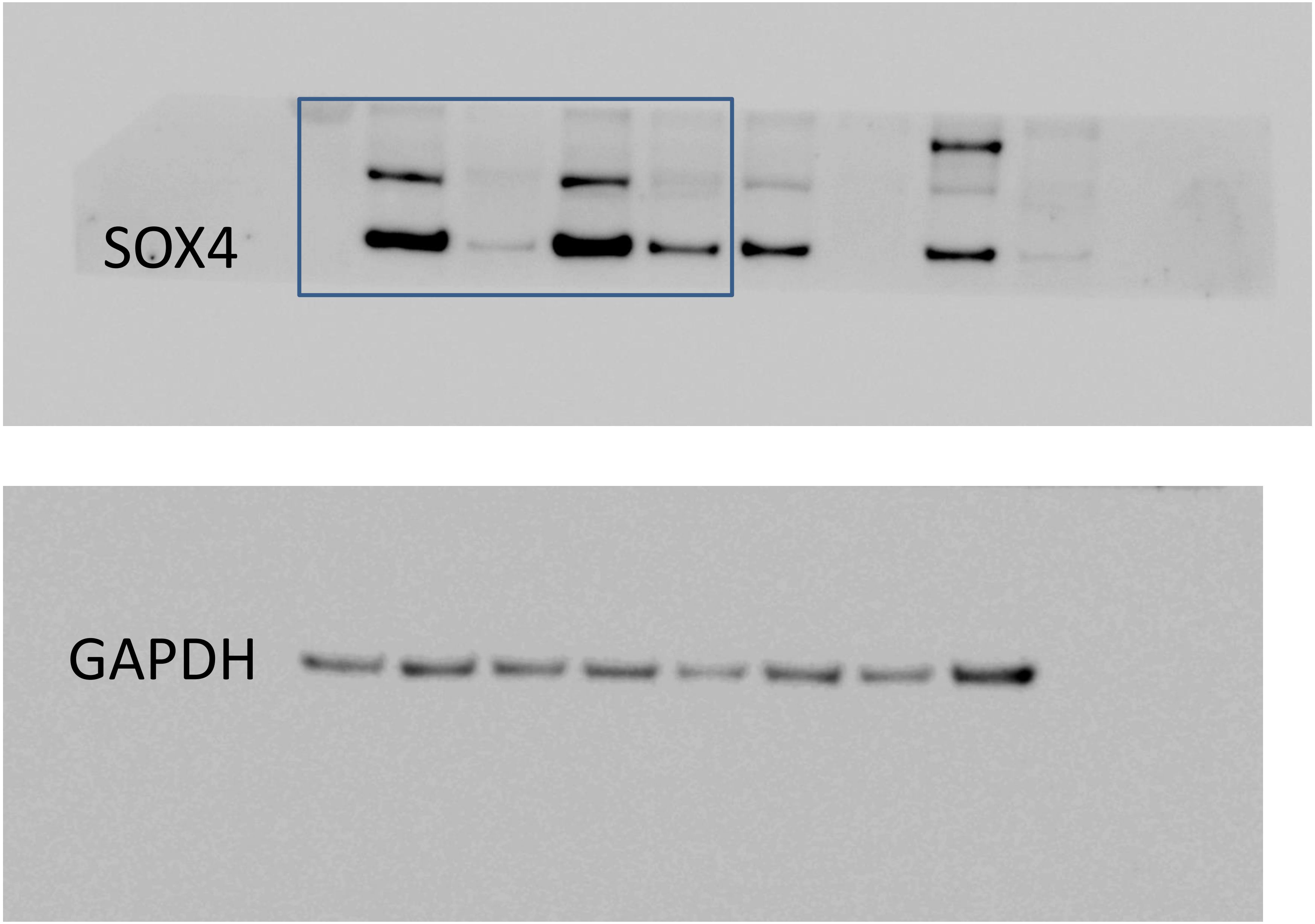

Figure 5E

SOX4 47kDa

GAPDH
34 – 36 kDa

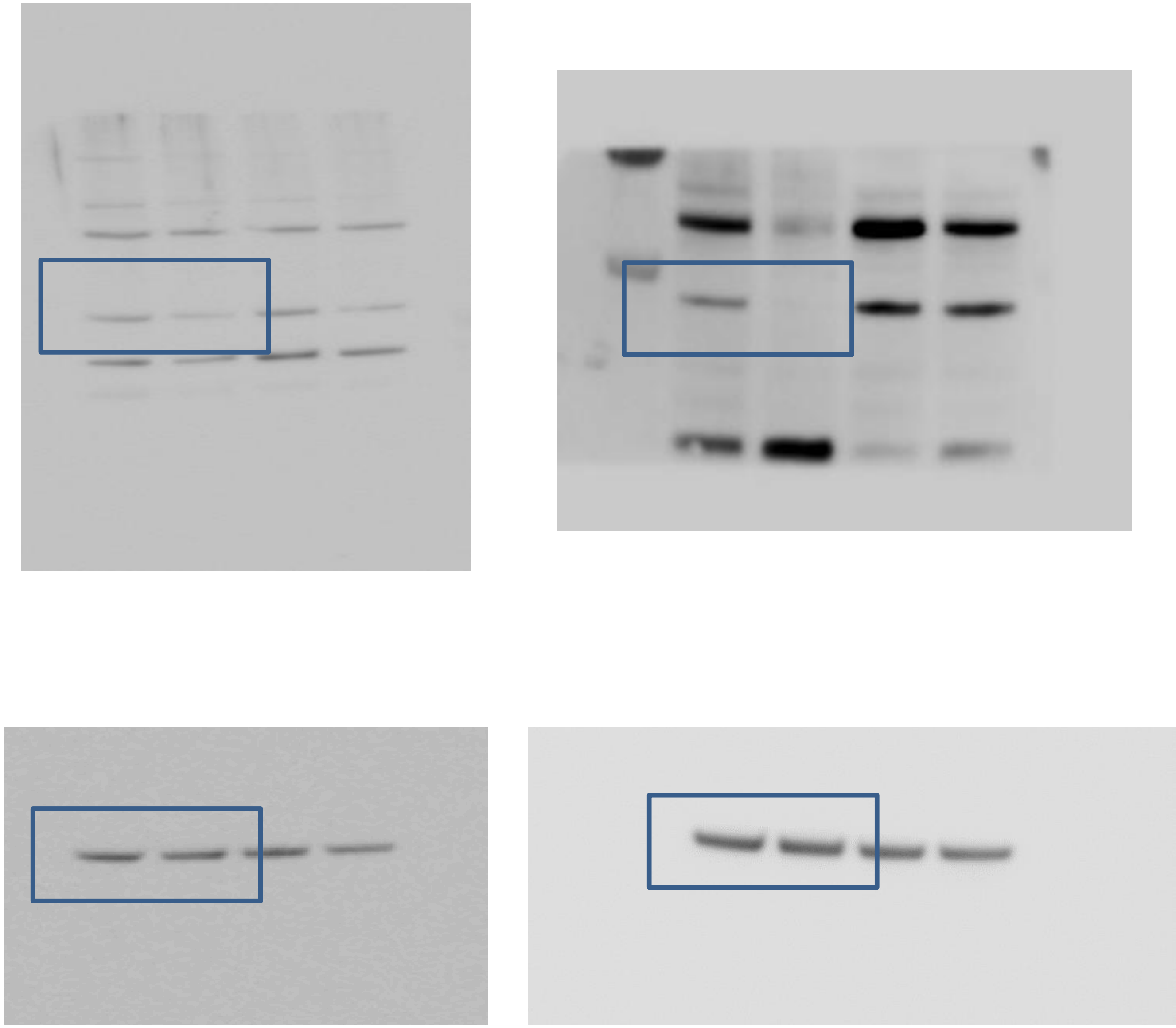

Figure 5F

A375 WM35

SOX4
47 kDa

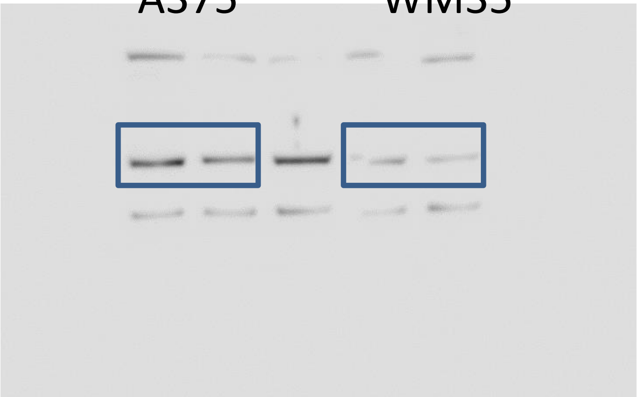

GAPDH
34-36 kDa

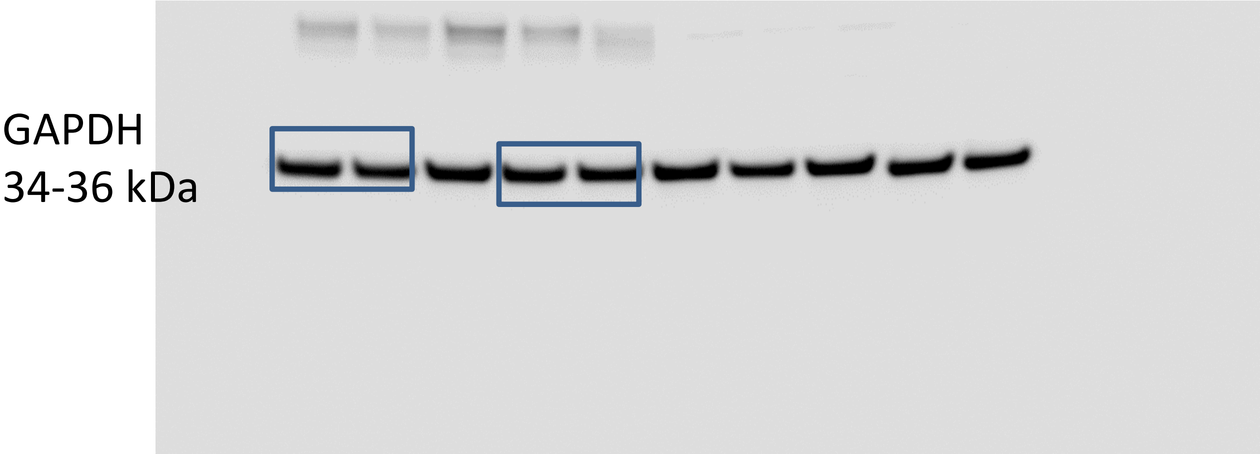

Figure 5H

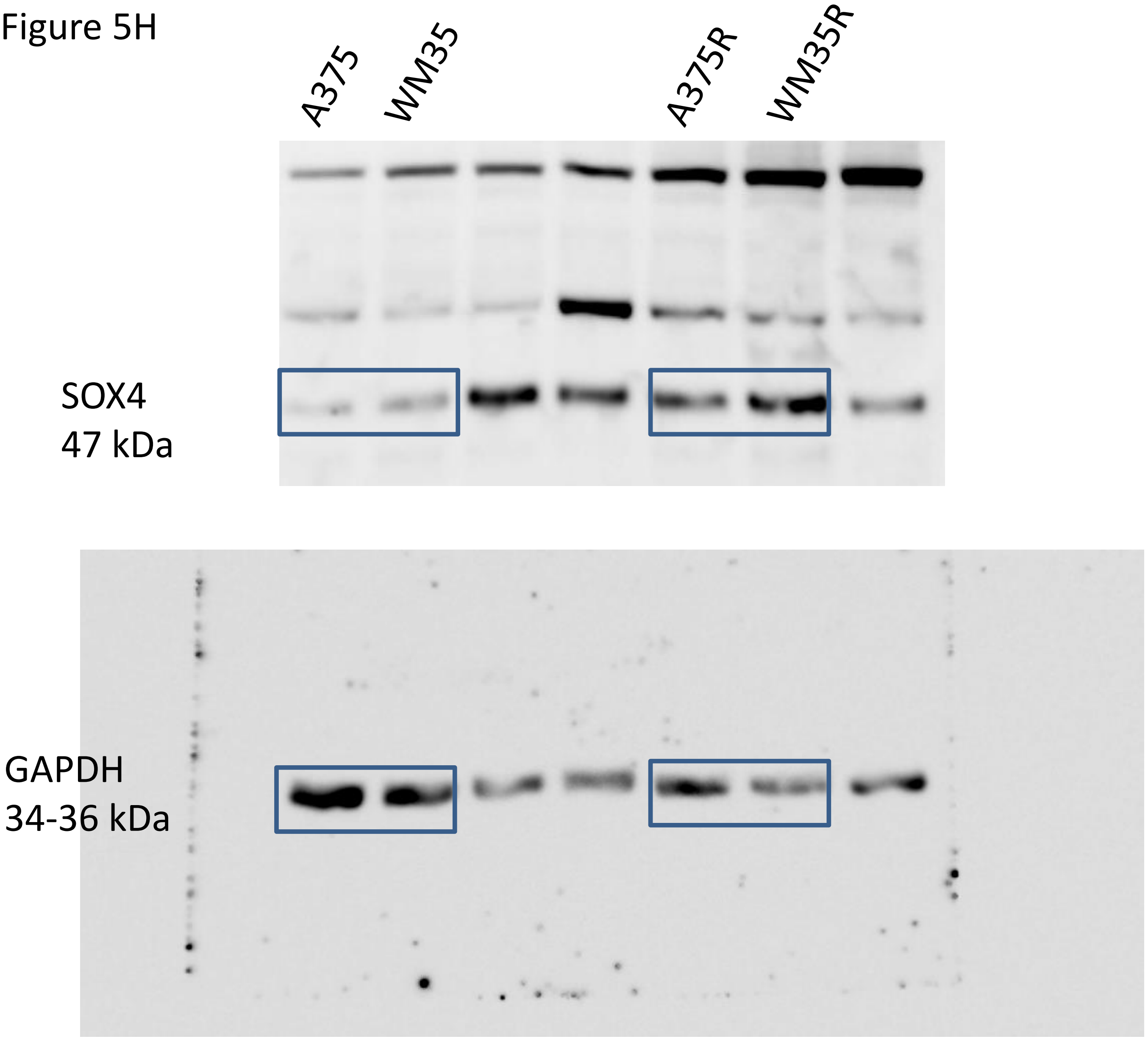

Supplemental figure 1

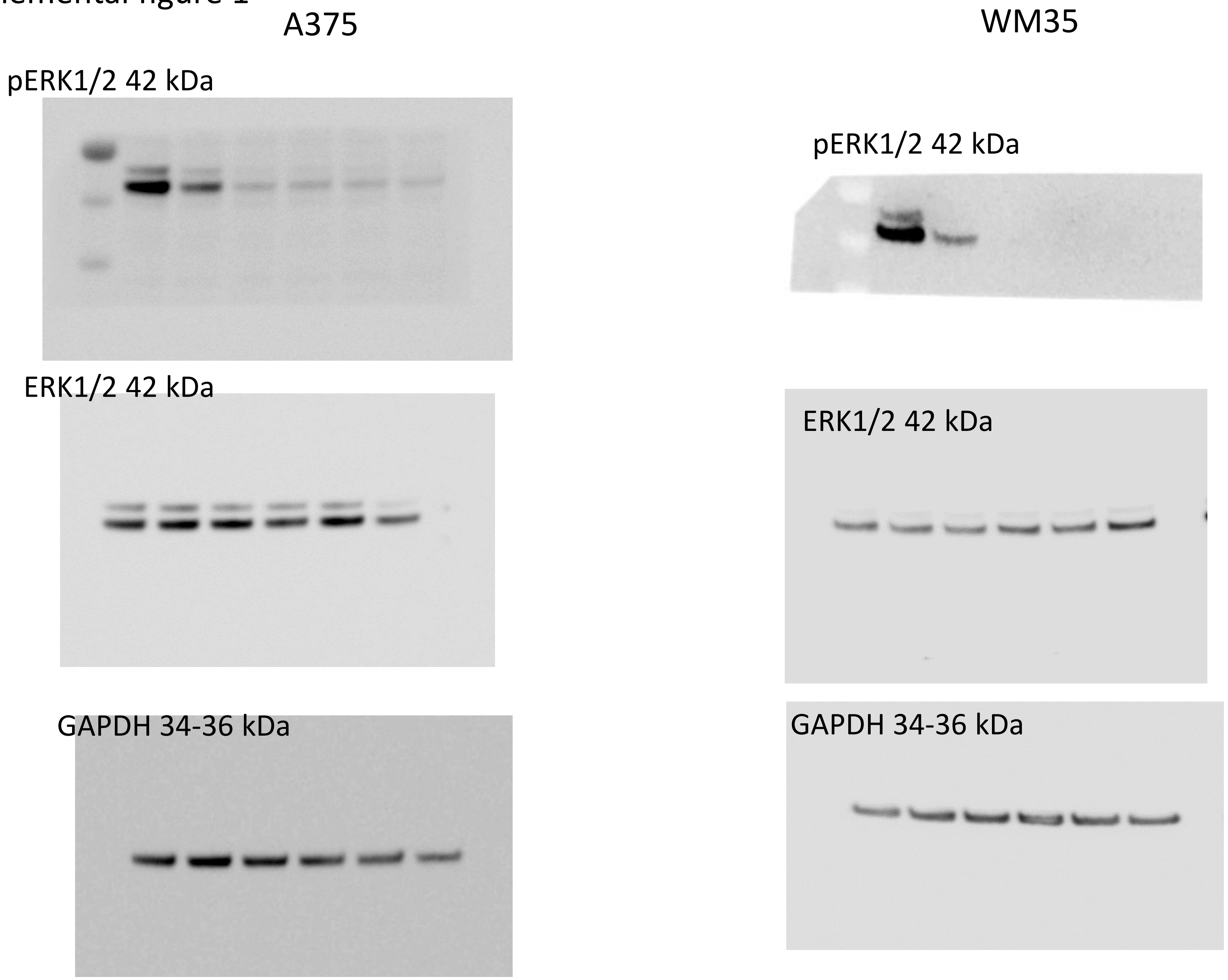

Supplemental figure 1

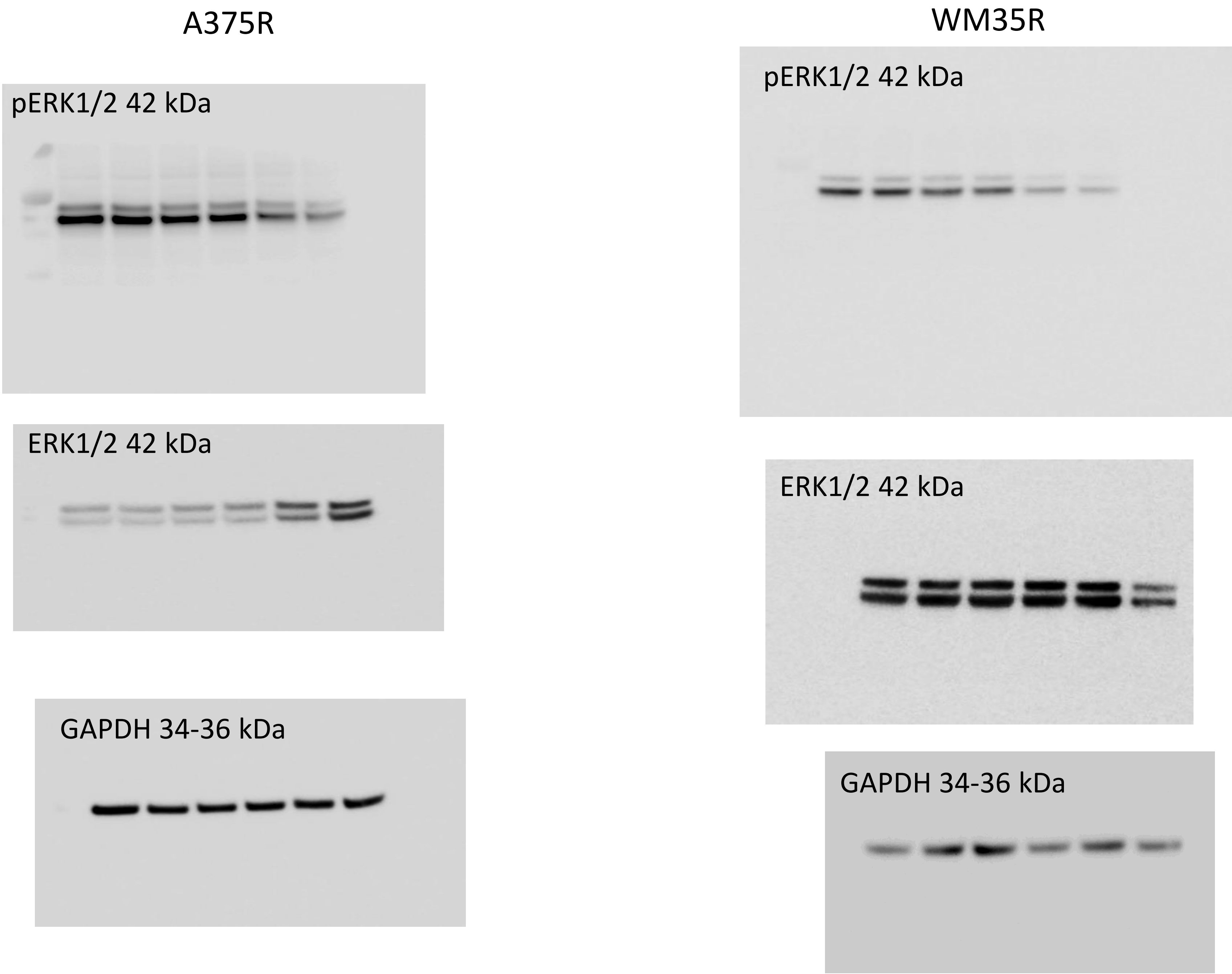

Supplemental Figure 3

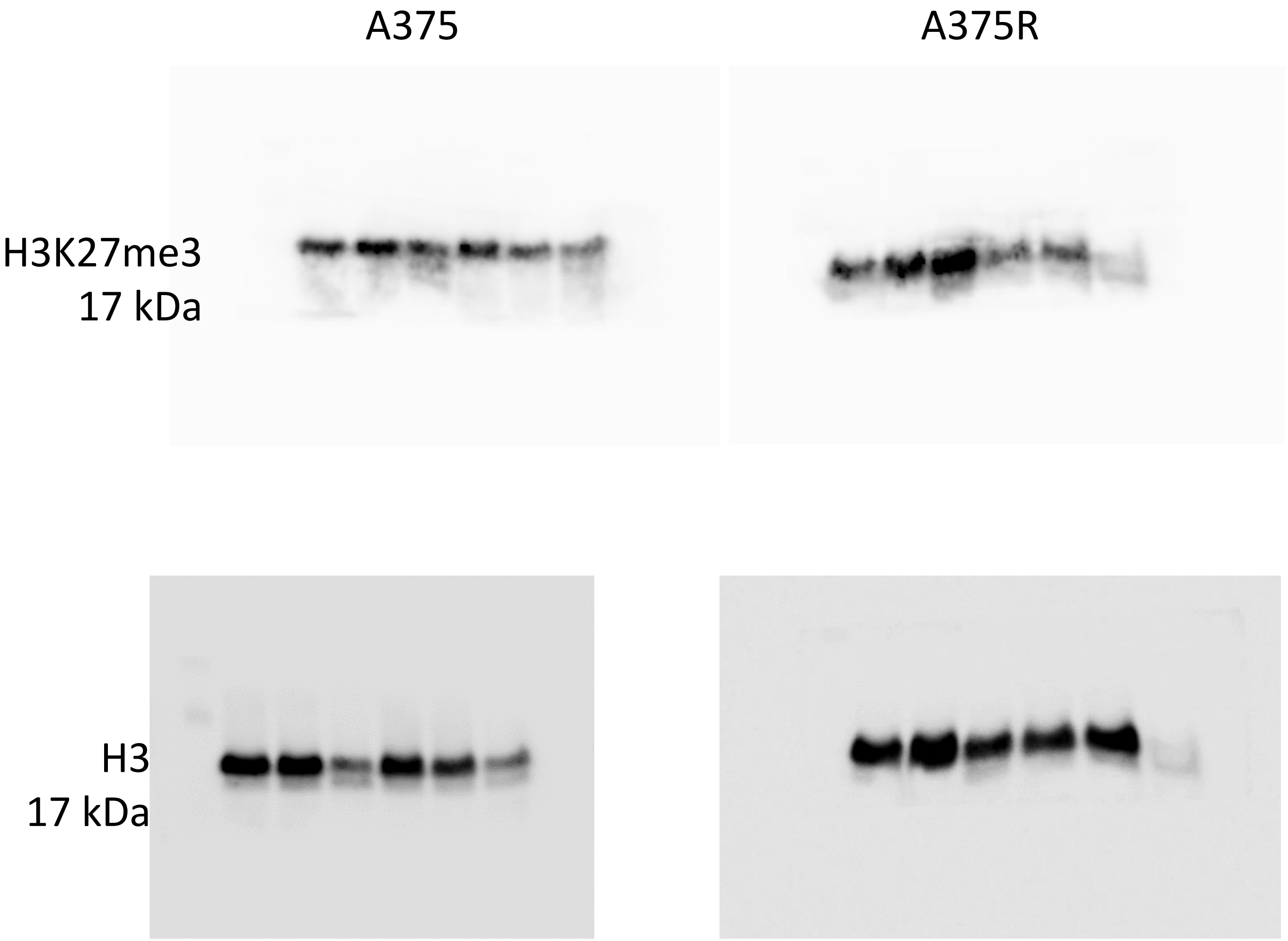

Supplemental Figure 3

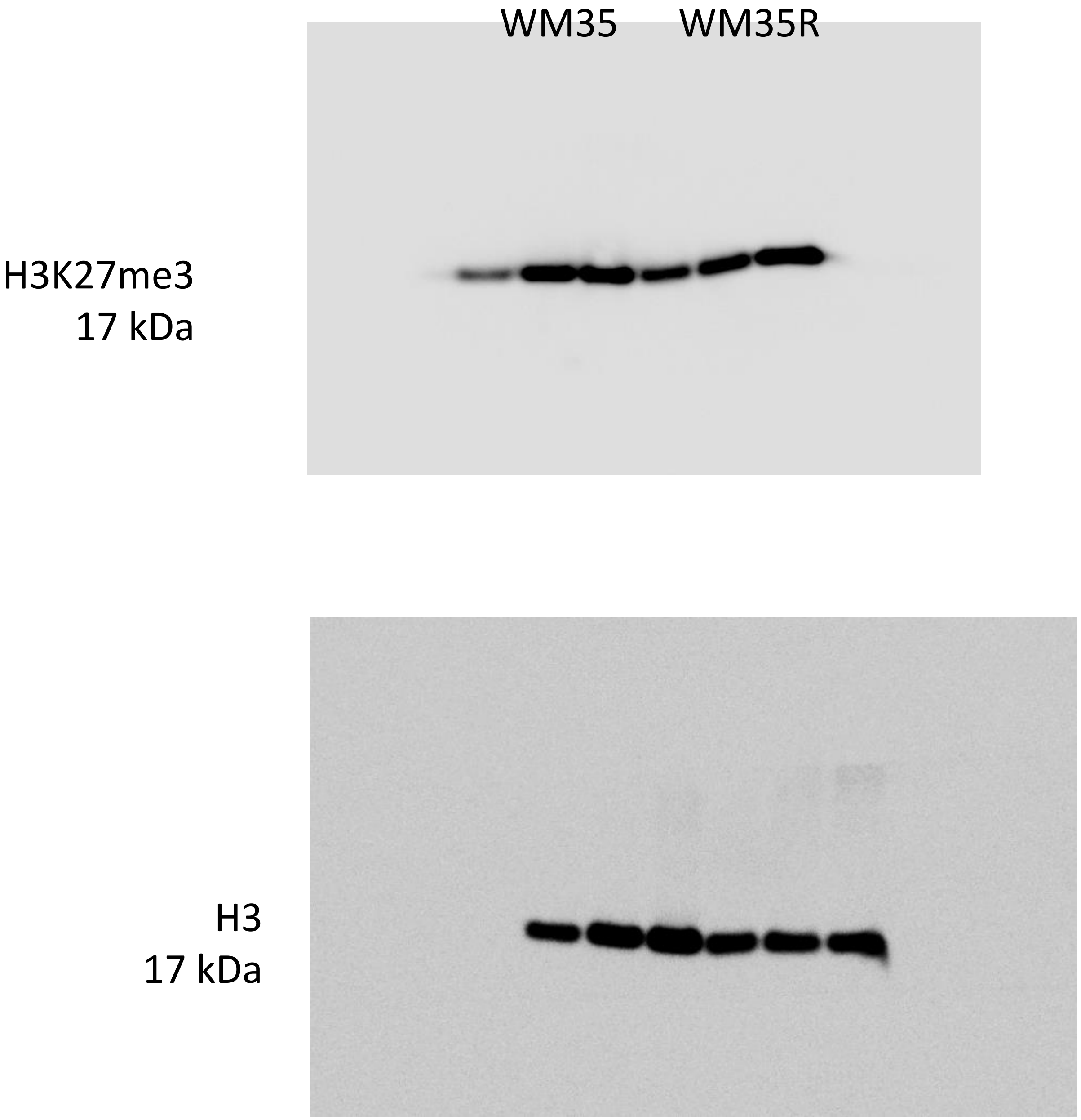

Supplement: Supplementary file 1 [file cancers-13-02393-s001.zip › Western blot originals.pdf]
